# Engineered Extracellular Vesicles as a Reliable Tool in Cancer Nanomedicine

**DOI:** 10.3390/cancers11121979

**Published:** 2019-12-09

**Authors:** Francesca Susa, Tania Limongi, Bianca Dumontel, Veronica Vighetto, Valentina Cauda

**Affiliations:** Department of Applied Science and Technology, Politecnico di Torino, Corso Duca degli Abruzzi 24, 10129 Turin, Italy; francesca.susa@polito.it (F.S.); tania.limongi@polito.it (T.L.); bianca.dumontel@polito.it (B.D.); veronica.vighetto@polito.it (V.V.)

**Keywords:** extracellular vesicles, exosomes, chemico-physical functionalization, loading, cancer, nanomedicine, translational medicine, nanotechnology: bioengineering

## Abstract

Fast diagnosis and more efficient therapies for cancer surely represent one of the huge tasks for the worldwide researchers’ and clinicians’ community. In the last two decades, our understanding of the biology and molecular pathology of cancer mechanisms, coupled with the continuous development of the material science and technological compounds, have successfully improved nanomedicine applications in oncology. This review argues on nanomedicine application of engineered extracellular vesicles (EVs) in oncology. All the most innovative processes of EVs engineering are discussed together with the related degree of applicability for each one of them in cancer nanomedicines.

## 1. Introduction

The latest literature reports underline that extracellular vesicles (EVs), released by prokaryotic and eukaryotes cells into the extracellular surroundings, are the main drivers of the intracellular communication, not only in physiological but also under pathological conditions [1,2,3,4,5,6,7,8,9]. 

The International Society for Extracellular Vesicles (ISEV) defines EVs generally as lipid bilayer-delimited particles released from cells and unable to replicate [10]. Agreement has not yet been reached on the specific markers for defining EVs subtypes, such as exosomes and ectosomes, originated from the endosome and the plasma membrane, respectively. Researchers are advised to contemplate the use of operational terms for EV subtype definition, referring to EVs’ physical characteristics such as size (<100 nm for “small EVs”, and > 200 nm for “medium/large EVs”), density, biochemical composition (tetraspanin/Annexin presence, etc.) and reference to condition or tissue/cell biogenesis (podocyte EVs, cardiosomes and prosatosomes, large oncosomes, apoptotic bodies) [10,11,12]. More in general, referring to their dimension and biogenesis’ mechanisms, EVs can be grouped into three broad categories: apoptotic bodies, ectosomes and exosomes [13,14].

Apoptotic bodies (ApoBDs) are typically 1–5 μm EVs released as cells’ blebs during the apoptotic process. They contain cytoplasm, organelles and often also nuclear fragment, lipids, proteins [15] and a high amounts of phosphatidylserine [16]. 

Ectosomes and exosomes formation rests on confined microdomains assembled in the plasma membrane for ectosomes and in the endocytic membrane system for exosomes [17]. Ectosomes (100–500 nm diameter) are larger than exosomes (30–150 nm diameter) and both their cargoes and membranes composition partially differ from each other. Exosomes originate from the endosomal compartment inside multivesicular bodies and they are released by the fusion with the plasma membrane. Exosomes’ membranes are rich in tetraspanins (CD9, CD63, CD81, CD82 and CD151) [18], sphingomyelin, cholesterol [19] and adhesion molecule (ICAM-1), while the ectosomes’ ones are characterized by plentiful of glycoproteins, receptors and metallo proteinases [17,20]. 

Oncosomes are exceptionally large ectosomes, typical of advanced cancers containing active molecules involved in the metabolic pathways promoting tumoral cell survival and growth [21].

Starting from the key role that the tumor microenvironment plays in cancer establishment and progression, it is easy to understand how the EVs have an active part in influencing processes as pre-metastatic niche development, oncogenic transfer, and immune modulation [22,23].

Tumor-derived EVs, by carrying chemokines, are able to induce white blood cells’ chemotactic response [24]. Tumor-derived exosomes promote inflammation compromising natural immunity and reprogramming T cells [25], while ApoBDs join in the horizontal oncogenes transfer thanks to the nuclear material that comes out from the dying cells by which they were produced [26]. 

Since EVs have an active role in the tumoral intercellular communication and signal transduction systems, it spontaneously comes out to consider their applications as biomarkers and therapeutic agents in oncology.

**Figure 1:**
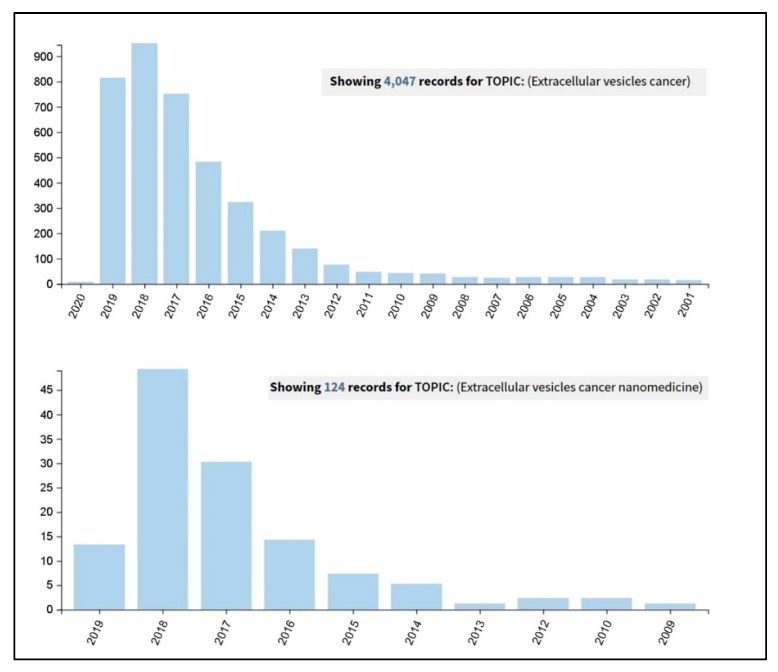
Results coming out from a Web of Science search carried out on the 26th September 2019, at the all databases level, for the terms ‘extracellular vesicles cancer’ (upper panel) and ‘extracellular vesicles cancer nanomedicine’ (lower panel).

It actually results very interesting to observe how an advanced Web of Science search (carried out on the 26th September 2019 at the all databases level) for the terms ‘extracellular vesicles cancer’ and ‘extracellular vesicles cancer nanomedicine’ has clearly shown an incredible increase in the number of publications in the last five years (Figure 1). A further more detailed analysis was carried out on these results and considered the percentages of the papers’ distribution in the various research areas. It revealed that, by adding the term ‘nanomedicine’ to the query, the percentage of papers in the section ‘Science technology other topics’ increases from the 25% to the 85%, thus demonstrating the current interdisciplinary research trend of this topic. 

Current trends refer to EVs as successfully non-invasive diagnostic and prognostic biomarkers: actually their membrane proteins, their lipid fingerprint (reflecting the protein and lipidic content of the parent cells at the moment of their formation) and micro RNA load can be easily screened in blood, urine and in other biological fluids [20,26,27]. 

Regarding EVs’ application as cancer therapeutics, it basically differs from conventional approaches, i.e., molecular targeting drugs and chemotherapy. Referring to native EVs, a huge number of in vivo and in vitro studies have been reported [28,29,30,31,32,33,34]. In details, three main approaches in cancer treatment through native EVs can be identified: the inhibition of EVs production [35,36,37], the eradication of circulating EVs, and finally the reduction of EV uptake [38,39].

**Figure 2:**
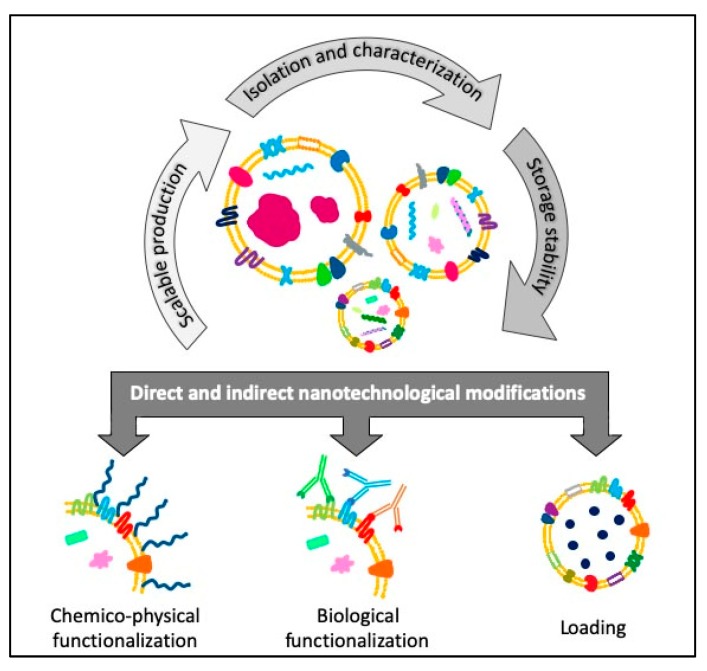
Schematic view of the flow of the different steps concerning the direct and indirect engineering of extracellular vesicles (EVs) for cancer diagnosis and therapy applications.

EVs are usually biocompatible, low immunogenic and non-cytotoxic, with a high loading ability, long life span in circulation and the capacity to cross barriers, i.e., the cytoplasmic and the blood brain barriers, making them suitable for drug delivery applications [40,41]. Furthermore, EVs are internalized 10 times more than liposomes of similar size in cancer cells, showing a higher specificity towards tumoral cells [42] and, thanks to their dimensions, they can also exploit the enhanced retention and permeability effect to accumulate in the cancerous tissues and reach easily the bulk of a solid tumor [43]. The research on EVs is making great strides in cancer medicine and there are already 136 clinical trials on exosomes and 36 on EVs listed on “www.clinicaltrials.gov” both for therapy and diagnosis. Given these premises, EVs can be considered promising tools for the development of new engineered devices for therapeutic and diagnostic applications. Starting from scalable, reproducible and well standardized EVs isolation procedures, it is possible to obtain highly purified products ready for further microscopic, immunological characterizations or for cryopreservation treatments able to guarantee the stability and integrity necessary for long-term storage or subsequent modifications. Otherwise, these modifications can be carried out directly by engineering the parent cells before the isolation, to obtain already loaded or functionalized EVs (Figure 2). 

In this review we summarize the last studies about the direct and indirect engineering of EVs. The first one takes place immediately after the isolation or thawing steps, by means of membrane permeabilization, surface functionalization or loading strategy. The second engineering method, i.e., the indirect one, happens when the engineering process is applied through molecular or genetic strategies on the parent cell that will secrete the vesicles.

## 2. EVs’ Post Isolation Direct Engineering 

### 2.1. Chemico-Physical Functionalization

Post-isolation modification techniques, enabling the functionalization of the EVs surface with specific moieties, improve their targeting abilities and biodistribution, allowing their in vivo and in vitro tracking.

The methods for the direct engineering of EVs surfaces can be essentially divided in covalent and non-covalent chemical modifications. In the first case, chemical reactions are performed between functionalizing molecules or chemical linker and the amine groups, which are reactive functional units widely expressed on exosomes’ surfaces [44]. Even if EVs, as non-living entities, have major advantages with respect to cells regarding reagents and reaction conditions, these must be carefully controlled and optimized in order to avoid vesicles disruption, denaturation and aggregation due to the use of inappropriate temperature, pressure, and/or osmolarity [16].

The non-covalent approaches, instead, refer to membrane modifications by milder reactions, based on electrostatic interactions and receptor-ligand binding as well as lipid-conjugated compounds post-insertion into the EVs’ lipid bilayer [44].

Fluorescent and magnetic labeling represent a couple of the main results of the research on EV surface modifications [44]. In fact, tracing the cellular trafficking of autologous exosomes or their biodistribution and pharmacokinetics is essential to investigate their possible diagnostic and therapeutic applications [45].

EVs can be efficiently labelled after their isolation with organic fluorescent dyes. This class of dyes is widely used for in vivo and in vitro imaging [45] and includes a variety of fluorophore-conjugates. These are able to selectively interact with different components of EVs, like RNA and DNA contained inside them [46] or directly with their lipid bilayer [47,48] or with the amine groups of surface proteins by covalent bonds [49].

Click-chemistry is successfully used for the functionalization of exosomes with fluorescent, radioactive and magnetic resonance imaging (MRI) agents for precise in vivo exosomes tracking [50]. In particular, exosomes are chemically modified with terminal alkyne groups by cross-linking the amine groups of exosomal membrane and the carboxyl group of 4-pentynoic acid using carbodiimide activation. In a second step, the inserted alkyne terminal groups are reacted with azide-fluor 545 to form a triazole linkage, according to the typical click-chemistry reaction [51]. In this way the number of cross-linked alkyne groups is controlled in order to avoid the overmodification of exosomal membrane proteins. With a standard calibration curve, it is estimated that approximately 1.5 alkyne modifications are made for every 150 kDa of exosomal protein [50], ensuring the preservation of size of exosomes and their capability of interaction with recipient cells.

The modification with polyethylene glycol (PEG) is a common approach used to prevent opsonization and extend the circulation half-life of liposomes and synthetic nanoparticles (NPs), it has also been successfully transferred to EVs. PEGylation of EVs results in a significant increase in circulation time after intravenous injection in mice, from 10 min for unmodified EVs to 60 min or even 240 min for PEG-functionalized exosomes. The decoration of EVs membrane is obtained, at 40 °C, by a post-insertion mechanism, combining PEG-phospholipids micelles (i.e., 1,2-dimyristoyl-sn-glycero-3-phosphoethanolamine (DMPE)-PEG) with EVs extracted from mouse neuroblastoma cells, maximizing the moieties incorporation while preserving EVs characteristics [52].

While opportunely minimizing the recognition by mononuclear phagocytic system, the PEG corona strongly reduces the EVs-cell interaction in vitro [52]. The further functionalization of distal end of PEG chain with appropriate targeting ligands, as already described for synthetic particles [53], can easily overcome this drawback and create a promising tool for drug delivery with stealth properties and targeting abilities. A very recent study [54] described how modify EVs’ surface with the active targeting ligand mannose. Exosomes’ surface was successfully modified with PEG, avoiding particle aggregation, through the incorporation of 1,2-distearoyl-sn-glycero-3-phosphoethanolamine (DSPE) into the lipid layer of the exosome. For targeting purposes, the PEG’s distal end, functionalized with amine groups, was further conjugated with mannose-isothiocyanate, guaranteeing a better accumulation of functional exosomes in lymph-nodes [54].

Exosomes loaded with paclitaxel (PTX) are modified with aminoethyl anisamide-polyethylene glycol (AA-PEG) as targeting ligand toward sigma receptor, overexpressed by lung cancer cells [55]. The AA-PEG complex has been inserted in exosomal membrane conjugated with DSPE lipid by using a process that includes sonication and incubation steps, already developed by the same laboratory for the drug loading [56]. The in vitro and in vivo uptake tests, confirmed that AA-PEG exosomes are taken up in much higher quantities than non-vectorized ones. Furthermore, the in vitro uptake of PEGylated exosomes without targeting moiety is lower than that of unmodified exosomes, probably due to the PEG chains blocking interaction of exosomal surface proteins [55].

The same principle of post-insertion of a lipid linked with a molecule able to provide a conjugation site for targeting ligands is used to functionalize exosomes membranes with folate and two RNA aptamers, specific for typical cancer receptors (i.e., prostate-specific membrane antigen (PSMA) RNA aptamer, and epidermal growth-factor receptor (EGFR) RNA aptamer) [57]. A cholesterol-triethylene glycol (TEG) is conjugated with the engineered packaging RNA-three-ways junction (pRNA-3WJ), exploiting the spontaneous insertion of the cholesterol via its hydrophobic moiety into the lipidic bilayer and thus able to anchor the 3WJ into the EVs membrane. The particular spatial conformation of the conjugate, with the cholesterol specifically placed on the arrow tail of the 3WJ, prevented the RNA ligand from trafficking into the EVs, ensuring an oriented surface display of targeting ligands for cancer receptor binding [57].

Enhancement of exosomes’ therapeutic ability has been also obtained by an electrostatic interaction of original exosomes and cationized pullulan [58]. Nakase et al. used cationic lipids to increase the exosomal surface charge and help the interaction between EVs and target cells [59]. The membrane charge modification is obtained by the use of lipofectamine (LTX), a commercially available transfection reagent containing cationic lipids, which adsorbs on the exosomes surface and can help the interaction with negatively charged cells surface. The positive charge conferred from cationic lipids is also exploited to functionalize the exosomes surface with a negative charged pH-sensitive fusogenic peptide, GALA, able to guarantee an effective intracellular fusion of exosomal and endosomal membranes and the subsequent cytosolic release of the exosomal contents [60], fundamental for efficient therapeutic applications. This double membrane functionalization, based on electrostatic interactions and GALA peptide, provides an enhanced cellular uptake and the cytosolic release of artificially encapsulated cargo in the treatment of cancerous Hela cells in vitro [59].

It is well known that glycosylation has an important role in different biological function of EVs, like in the cargo proteins recruitment [61] and in the cellular recognition and uptake [62]. Recent glycomic analyses [63], performed by lectin microarray technology on EVs derived from different biological sources, reveal both enrichment and exclusion of glycan epitopes with respect to the membranes of their parental cells. In general lectin analyses reveal that EVs are enriched in high mannose, complex N-linked glycan, poly N-acetyllactosamine epitopes and in α-2,6 sialic acid [63], which is certainly involved in exosomes-cells interaction, thanks to sialic acid-recognizing lectins present on cell surfaces [64].

Furthermore, alteration in glycosylation pattern has been associated with different pathologies, including cancer [65], in which glycan changes took a variety of forms, i.e., loss or excessive expression of certain glycans, increased expression of incomplete or truncated glycans and, less commonly, appearance of novel glycans [66]. These changes are non-random, but closely correlated with malignant transformation and progression [66], making glycan structures valuable targets for anti-tumoral diagnostic and therapeutic strategies. 

Direct manipulation of glycosylation could be used to modify the surface of EVs in order to obtain enhanced delivery or specific targeting to selected tissues for therapeutic purposes. In a recent study, modified EVs are produced by treating them with an enzyme (neuraminidase) able to digest the glycoproteins’ terminal sialic acid residues [67]. The glycosidase treatment produces different in vivo biodistribution, causing for example a better accumulation in axillary lymph nodes of modified EVs compared to untreated ones, suggesting their valuable application as drug carriers when the lymphatic system is targeted [67].

A similar enzymatic treatment with a pan-sialic acid hydrolase is performed to reduce the expression of immune inhibitory sialic acids on glioblastoma-derived EVs [68]. Lectin-binding analyses confirmed that surface glycoconjugates of glioblastoma-derived EVs are dominated by immune inhibitory sialic acid-capped N-glycans and complex bi-antennary glycans [69].

Thus, manipulation of surface glycosylation combined with the insertion of a high affinity ligand for DC-specific ICAM-3-grabbing non-integrin (DC-SIGN) receptor leads to an enhanced internalization of glioblastoma-derived EVs in dendritic cells for the triggering of an efficient anti-tumor immune response [68].

Another valuable application of protein glycoengineering could be found in the stabilization of targeting peptides fused to exosomal membrane protein. It was demonstrated that peptides expressed on the N-terminus of lysosome-associated membrane glycoprotein 2b (Lamp2b) could be degraded during exosomes biogenesis by endosomal proteases [70]. The inclusion of a glycosylation motif to the N-terminus of the fusion protein efficiently protects the targeting peptide from proteolysis, enhancing its expression in exosomes membrane while preserving the peptide-target interactions [70].

Other post-isolation functionalization techniques involve biological molecules as receptors or antigens. A33 antigen has been proven to be overexpressed in colorectal cancer cells, demonstrating to be a novel target as immunotherapeutic agent for cancer therapy even in clinical trials (NCT00003360, NCT00199862 and NCT00291486). EVs isolated from colorectal cancer cell line present the A33 antigen on their surface. These EVs are loaded with doxorubicin and functionalized with superparamagnetic iron oxide nanoparticles (SPIONs) coated with high-density A33 antibodies, forming a complex with antitumor activity towards colorectal cancer with reduced systemic toxicity [71]. 

EVs have been post-extraction engineered also for the treatment of glioblastoma multiforme. Methotrexate-loaded EVs were functionalized with the pro-apoptotic peptide KLA, and the targeted peptide, low-density lipoprotein (LDL), to target the LDL receptor overexpressed on the blood brain barrier and glioblastoma cells [72]. A summary of the post-extraction chemico-physical modifications of EVs is presented in Table 1.

### 2.2. Loading Nanotechnological Modification into EVs

EVs can be successfully engineered acting as vehicles to transport different types of cargo such as drugs, active molecules, nucleic acids and nanoparticles for imaging, tracking or therapeutic purposes in cancer biology or medicine [78,79].

As widely reported in the literature [16,80], several methods are used to incorporate cargoes inside EVs. Exogenous methods for loading EVs require the isolation of the vesicles at first, and their successively loading according to different procedures. 

One of the simplest way is to co-incubate the EVs with the desired content, which will penetrate into the vesicle membrane due to the different gradient of its concentration between the two sides of the EVs membrane [81,82]. In particular, in the case of hydrophobic compounds, the internalization could be reached by a simple passive diffusion process. The lipidic membrane presents a hydrophobic region completely separated from the intra and the extra cellular region, thus incubation with high concentration of drugs, such as doxorubicin, paclitaxel or imatinib, generates EVs loaded with therapeutics molecules [47,83,84]. This strategy requires minimal manipulations and allows the loading of a high amount of molecules [85].

Otherwise, the loading of hydrophilic molecules inside the intraluminal space requires the mechanical or chemical disruption of the lipid envelope. Electroporation is based on the application of an electric field to the EVs solution to create nanosized pores in the vesicles’ phospholipidic membrane, enabling the diffusion of the desired drug [59,79,86], small interfering DNA (siRNA) [87,88], DNA [89] or NPs [90], maintaining the biological activity of the cargo. However, electroporation can change the physical characteristics of EVs and it is applicable only to small molecules, which can also aggregate and stick on the EVs’ surface [85]. 

A sequence of freeze-thawing cycles of the EVs, which leads to the disruption of their membranes, can be used as exogenous method of loading. Membrane fractures or deformations, due to the ice-crystals formation, induce the encapsulation of relatively bulky molecules [91] such as proteins [92] and nanoparticles [93] without affecting their biological activity.

Another method of loading hydrophilic compounds inside EVs is sonication: vesicles and exogenous molecules can be mixed together and exposed to ultrasound. By disrupting the lipid membranes, the incorporation of the molecules inside the EVs occurs while the membrane is auto reconstructing [94]. This method prevents the aggregation of sensitive cargoes such as siRNA [95]. Nevertheless, both sonication and freeze-thawing methods cause a significant increase in the size of EVs, indicating that their morphofunctional characteristics could be in some way compromised [56,92,96].

EVs and cargo molecules can be also incubated together and then extruded through the use of a syringe-based lipid extruder. The process disrupts the vesicle membrane allowing the mixing of the different components in solution [97]. This method is recognized as the most efficient loading technique for water soluble cargoes such as many anticancer drugs and catalase are [98,99].

EVs’ membranes permeabilization can be achieved also through chemicals stimuli: detergents like saponin can dissolve cholesterol forming pores in the membrane, altering its permeability and allowing the cargo penetration inside the vesicles [97,100]. 

The possibility of being packaged in extracellular vesicles could represent a great plus in oncology also for a wide range of just Food and Drug Administration (FDA)-approved drugs. Paclitaxel (PTX) is a potent chemotherapeutic agent, used in multi drug resistant (MDR) cancer treatments. Its encapsulation into EVs has been largely studied to increase the efficiency of cargo release and the preferential accumulation into cancer cells, suggesting the possibility to obtain a higher targeting specificity [47,56,101]. PTX encapsulated in endothelial cell-derived exosomes has the ability to cross the BBB and be released in brain tumors in vivo [81]. PTX can be also encapsulated in milk-derived EVs which improve the efficacy of the drug and decrease the immunologic toxicity [41,102]. 

Doxorubicin (DOX) is an antineoplastic drug used for the treatment of different cancers, such as breast cancer, leukemia, lymphoma. Its cardiotoxicity, limits its applications and its maximum tolerated dose, thus a delivery vehicle is needed to improve DOX biodistribution. A wide number of studies have been done on the encapsulation of doxorubicin in EVs [83,103,104]. The efficiency of the EVs-DOX nanoconstruct has been validated both in vitro and in vivo, as vehicle for a targeting delivery of the drug to breast cancer cells, reducing the relative side effects [79,105,106]. The encapsulation in EVs drastically reduced the in vivo cardiotoxicity of free DOX and the accumulation of the drug in cardiac tissues is diminished of approximatively the 40%, without affecting the efficacy of DOX towards cancerous cells [107]. 

Curcumin is another drug widely used in clinics with a variety of applications, due to its antioxidant and anti-inflammatory properties [108]. While curcumin does not show any antitumor properties when administered via dietary, it reveals a significant inhibition of tumor growth in vivo when loaded into exosome [109]. Another property of curcumin, loaded in EVs, is the partial reversal inhibition of NK cell tumor cytotoxicity in breast tumor cells, supporting the anti-cancer behavior of this bio-system [110]. The safety and efficacy of this system has been largely confirmed, leading to clinical trials. From 2011 plant exosomes have been used to deliver curcumin in clinical trial (NCT01294072) to treat colon cancer tissue.

Another category of cargo is composed by biomolecules as siRNA, miRNA (microRNA or miR): siRNAs are considered as promising anticancer treatment, owing to the ability to modulate oncogene expression levels [111]. Challenges in the use of RNAs are their rapid clearance into the blood stream and its inability to cross cell membrane. In this contest EVs could represent are a promising vehicle to deliver therapeutic RNA, due to their carrying ability and to their affinity with cell’s membrane.

Considering the various types of materials that can be loaded into the vesicles with the techniques described above, we can assert that NPs can be finely tuned for a wide number of applications through a strict control of physical and chemical parameters [112]. However, when administered into the body, they could face some problems such as opsonization and/or aggregation [113,114]. 

The first phenomenon could lead to their recognition and elimination by the immune system, while the second one could cause deleterious effects, such as thrombosis and accumulation in off-target organs as liver, spleen and kidney. The shielding of NPs with EVs can thus potentially help to overcome these drawbacks: the biomimetic coating can prevent or reduce the aspecific interaction with proteins and, if further functionalized with targeting molecules, can avoid NPs’ accumulation in undesired tissues and organs [115,116,117].

Internalization of gold NPs into exosome has been largely studied for different purposes, such as therapeutic and diagnostic ones [78]. The combination of gold NPs loaded with doxorubicin and encapsulated in EVs decreases drug’s toxicity increasing its delivery to the cancer cells [118]. 

Metal-organic frameworks (MOF) NPs have recently emerged as valuable nanocarriers, due to their biocompatibility and high loading efficiency. However, cargo leakages and degradation before they reach their target cells must be avoided, and a biomimetic shield sometimes solves these problems. The encapsulation of MOF NPs inside exosomes, achieved by simple incubation, allows the delivery of the anticancer drug as suberoyl bishydroxamic acid [82] or the protein gelonin [119] able to target cancer cells, avoiding the premature cargo leakage and the degradation caused by the protease enzymes.

Another class of NPs broadly used in the biomedical field is the iron oxide one and, more specifically, SPIONs are known for their magnetic, imaging, and heating capabilities. Literature refers to EVs loaded with iron oxide nanoparticles and a clinical photosensitizer molecule (Foscan) used as biocamouflaged agents for photodynamic therapy, magnetic resonance imaging, magnetic manipulation, and hyperthermia [120]. Gold-iron oxide NPs covered with tumor cell-derived EVs are successfully indicated for theranostic applications, as they result suitable for both magnetic resonance imaging and photothermal treatment at the same time [121]. 

A new nanoconstruct exploits the peculiar features of zinc oxide NPs (ZnO NPs) to treat cancer without the addition of drugs. Zinc oxide NPs encapsulated inside the EVs can be efficiently internalized by cancer cells causing their apoptosis [122]. A summary of cargo-loaded EVs with the related bibliographic references is provided in Table 2.

## 3. EVs’ Indirect Nanotechnological Modification through Parent Cells’ Engineering

A frequently applied method to modify EVs in vitro, i.e., loading cargo molecules or accomplishing membrane functionalization, is through the engineering of parent cells. Cell engineering methods, such as genetic and metabolic modification and exogenous delivery, can alter the surface expression and cargo content of newly-produced EVs and thus enhance their biocompatibility, targeting and therapeutic abilities [129]. 

### 3.1. Indirect Surface Functionalization

EVs’ membrane is a complex structure constituted by phospholipids and membrane proteins. Since the membrane is the first point of contact with the cell, tuning its composition strongly improves the targeting ability and enhances the therapeutic ability of EVs [85]. 

This approach can be employed for the non-invasive monitoring of EVs in vivo exploiting the fluorescence of some binding molecules. Molecular imaging allows a quantification of the EVs biodistribution and, eventually, a therapeutic effect over the time. For instance, pancreatic cell lines that stably expresses the green fluorescent protein (GFP) linked to CD63 can produce exosomes consistently positive to GFP [130,131,132]. Another effective labelling strategy for EVs is the incorporation of an azido-sugar in the glycans through a combined metabolic glycan labelling click chemistry reaction. Tetraacetylated N-azidoacetyl-D-mannosamine (Ac4ManNAz) is placed in culture with the parent cells, spontaneously incorporated into glycans and uniformly redistributed on their EVs. The azido-EVs are then labeled with azadibenzylcyclooctyne (ADIBO)-fluorescent dyes by a bioorthogonal click reaction [133]. Exploiting the principle of bioluminescence for tracking EVs, in vivo Gaussian Luciferase (Gluc) is linked to a transmembrane domain of a platelet-derived growth factor receptor [134,135], or a lactadherin [136,137]. Gluc is the only naturally-produced luciferase that can emit flash of bioluminescence in the presence of oxygen as cofactor for the reaction. After the engineering of parent cells with Gluc, the produced EVs are extracted and, when administered systemically, they can be tracked in vivo thanks to their bioluminescence [134,135]. The cellular transgene expression into the parent cell allows the expression of the candidate protein or peptide in the released EVs. The coding sequence of the desired ligand is inserted by a gene transfer vector (i.e., lentivirus) between the signal peptide and the N-terminus of the mature peptide of a transmembrane protein. In this way, the parent cells can generate EVs with the peptide of interest on their surface [129]. The candidate protein or peptide, after the transfection in the parent cells, fuses with EVs membrane proteins such as Lamp2b and tetraspanins CD63 and CD9 [43], thus the produced EVs display the just-engineered molecule on their surface. For instance, dendritic cells can be engineered to express a protein composed by Lamp2b and αv integrin-specific iRGD peptide in order to secrete iRGD peptide-EVs. This functionalization considerably increases the delivery of doxorubicin to αv integrin-positive breast cancer cells in vitro [79]. The transfection can occur by using plasmid vectors. A plasmid vector encoding streptavidin (which binds to biotin with high affinity) and lactadherin (an exosome-tropic protein) fusion protein allow to obtain streptavidin-lactadherin-modified exosomes that are mixed with the biotinylated pH-sensitive fusogenic GALA peptide exerting a lytic activity in acid environment [138]. Lentiviral vector bearing LAMP2b-Designed ankyrin repeat protein (DARPin) G3 chimeric gene or herpes simplex virus with plasmid pACgp67B-HER2m, containing the anti-human epidermal growth factor receptor 2 (HER2) scFv (ML39) antibody DNA sequence, are used to engineer HEK-293T cells. EVs isolated from transfected cells can bind specifically to HER2/Neu in adenocarcinoma cell lines [139,140]. Human carcinoembryonic antigen or human HER2/neu can be also inserted into the mouse lactadherin expression plasmid p6mLC1C2 and transfected into dendritic cells, enhancing the production of functionalized EVs to target breast cancer cells [141]. Similarly, prostate-specific antigen and prostatic acid phosphatase linked to the C1C2 domain of lactadherin produce EVs that specifically target prostate cancer cells [142]. In another study, an anti-epidermal growth factor receptor (EGFR) nanobodies with anchor signal peptide glycosylphosphatidylinositol (GPI) fusion protein are transfected to parent cells in order to generate EVs with this functionalization. These EVs show a significantly improved targeting ability towards EGFR-positive tumor cells [143].

An alternative strategy is the hydrophobic insertion used to functionalize the EVs’ membranes by exploiting the phospholipid composition of plasma membranes. Amphiphilic molecule DSPE-PEG, FDA approved for medical applications, can self-assemble in the phospholipid bilayer [144]. Based on this consideration, if DSPE-PEG is bound to the molecule of interest, it can be incorporated inside the cell membrane, making it overexpresses the molecule on its surface and producing EVs with the desired functionalization. The most frequently used molecules are biotin and folate: the first one binds selectively with streptavidin, used for further functionalization, and the second one targets specific cancer cells [86,145,146,147]. In addition to folate, also other binding sites can be created on EVs using this approach, for example by adding the RGD sequence or sulfhydryl groups [148].

A summary of EVs ‘surface functionalization nanotechnological modification through parent cells’ engineering with the related bibliographic references is reported in Table 3.

### 3.2. Indirect Loading

Genetically engineered parent cells allow the production of pre-loaded EVs. This approach enhances the loading efficiency of molecules inside the EVs compared to the post-isolation techniques, minimizing the impairment of the structures or of the biological activity of both cargoes and carriers [44]. Some reports demonstrate the successful internalization of miRNA, siRNA [84,154] and nanoparticles [155] inside EVs produced from engineered parental cells. Furthermore, cells can be transfected with short RNA-encoding plasmid DNA (pDNA) in order to generate EVs enriched with target RNA [121,156]. The efficiency of cargo uptake inside EVs strongly depends on its high concentration inside the parent cells, because only a small amount is released as packed in the EVs [85]. Loading proteins inside the EVs can be accomplished by transfecting the parent cell with a vector containing the gene which codifies the specific protein. Proteins encoded by the transfected gene are synthesized by the cells and then secreted enveloped in EVs. Despite the apparent simplicity of this approach, many aspects need to be considered. The expression of cytotoxic proteins can inhibit the growth of the parent cells or induce their apoptosis. Furthermore, impaired biological reactions and interactions can obstacle the production of EVs ability [40]. 

Viruses are often used for the transfection of genetic materials or molecules inside the parent cells in vitro. Different kind of viruses are employed, but the most used is lentivirus because of its transfection ability and safety. Generally, the transfection of parent cells has the aim to overexpress a particular therapeutic or anticancer molecule in order to secrete it as enveloped inside EVs. For example, EVs-enriched human MUC1 (hMUC1) injected intra-dermally suppress the growth of hMUC1-expressing tumor [157]. Similarly, tumor necrosis factor (TNF)-related apoptosis-inducing ligand (TRAIL), a widely tested anticancer protein, causes the apoptosis of transformed or tumoral cells, but not of the normal ones. Due to its therapeutic efficacy, it has been encapsulated in EVs to overcome the shortcomings of a poor pharmacokinetic profile and the tumor resistance to drug [158,159]. Target proteins can also be delivered inside the parent cell by fusion with constitutive proteins of EVs, such as CD63, to improve the specificity of the protein loaded inside EVs [160]. Nef/E7 DNA vector expressing Nef exosome-anchoring protein combined with HPV-E7 is delivered to parent cells to make them able to generate immunogenic EVs containing the Nef-E7 fusion protein to elicit an efficient anti-E7 cytotoxic T lymphocyte immune response for cancer therapy [161]. Another strategy to incorporate proteins of interest inside EVs is pseudotyping, which packages viral RNAs or DNAs with the envelope proteins from another virus. The G glycoprotein of the vesicular stomatitis virus glycoprotein (VSVG) is frequently used for this purpose because of its efficacy in transduction and broad tropism. The selected protein is fused with VSVG and transfected into different parent cell lines [162]. This method can be further developed by adding to VSVG cell-recognizing peptides for targeting or engineered therapeutic antibodies, such as anti-CD19 chimeric antigen receptors, that target specific suppressors of cytotoxic T cells for cancer therapy [163]. A novel method, called EXPLORs (exosomes for protein loading via optically reversible protein-protein interactions), allows the loading of cargo proteins inside EVs through endogenous biogenesis processes, delivering soluble proteins into the cytosol via controlled, reversible protein-protein interactions. For this purpose, a photoreceptor cryptochrome 2 (CRY2) and CRY-interacting basic-helix-loop-helix 1 (CIB1) protein module, which regulates the floral initiation of Arabidopsis thaliana via blue light-dependent phosphorylation, are selected. Then, a transient docking of CRY2-conjugated cargo proteins is induced by introducing CIBN (a truncated version of CIB1) conjugated with an exosome-associated tetraspanin protein CD9 and by blue light illumination. After the release of the EVs with the cargo proteins linked to tetraspanins from the parent cell, they can be detached from CD9-conjugated CIBN by the removal of the illumination source, releasing them into the intraluminal space of the EVs [164]. 

The strategies described above to load EVs with proteins by engineering of the parent cells can be applied also in the case of nucleic acids. For instance, to reverse the chemoresistance to cisplatin-refractory gastric cancer, human embryonic kidney 293T (HEK-293T) cells are transfected with anti-miR-214 and the produced vesicles are administered systemically in combination with cisplatin, injected intraperitoneally, to overcome the in vitro and in vivo drug-resistance [165]. EVs produced by miR-134 or anti-miR-21 transfected mammary carcinoma cells have the ability to reduce the cellular proliferation and migration and to enhance the apoptosis in breast cancers [121,166]. miR-122 is essential to tune the chemosensitivity of hepatocellular carcinoma cells. Its effective delivery is accomplished by transfecting adipose-derived mesenchymal stem cells in order to produce EVs already loaded with miR-122 [167]. EVs from mesenchymal stem cells transfected with miR-146b expressing plasmid silence the EGFR and significantly decrease glioma growth [168], while EVs loaded with miR-143 inhibit the migration ability of osteosarcoma cells [169]. Mesenchymal stem cells can be loaded with anti-miR-9 to produce anti-miR-9 EVs. Anti-miR-9 delivered to cancer cells can reverse the expression of P-glycoprotein, involved in the chemoresistance, to enhance the efficacy of the temozolomide in otherwise resistant glioblastomas [170]. HEK-293T cell line can be genetically engineered to overexpress a suicide gene mRNA and protein-cytosine deaminase fused to uracil phosphoribosyltransferase in their microvesicles. They can transfer the therapeutic mRNA/protein to schwannoma cancer cells, achieving the inhibition of tumor growth [171]. Prostate cancer cell line is incubated with spherical nucleic acids (SNA), which are a new type of therapeutic agent composed by a core of gold nanoparticle with a dense shell of highly oriented nucleotides. The secreted EVs display a potent gene knockdown, when internalized in cancer cell, due to the presence of the anti-miR-21 [155]. EVs overexpressing hepatocyte growth factor (HGF) siRNA drastically reduced HGF and vascular endothelial growth factor (VEGF) expression in gastric cancer [172]. EVs delivery of siRNA against RAD51 and RAD52 causes an inhibition of proliferation and a massive reproductive cell death in human breast cancer cells [173].

The previously described method EXPLOR can be also used for the encapsulation of peptides inside cells, in particular of miR-21 sponges inside HEK-293T cells. The EVs produced are then loaded with this nucleic acid, which is an inhibitor of miR-21, overexpressed in most cancer types, and reduces the tumor progression and metastasis. After the collection of EVs loaded with miR-21 sponges, EVs are functionalized with cholesterol-AS411 aptamers exploiting the interaction with lipids of EVs’ membrane. The expression of AS1411 on EVs allows the targeting of leukemia cells for the interaction with nucleolin, overexpressed by these cancer lines. miR-21 sponges can inhibit miR-21 functions, triggering leukemia cells’ apoptosis [174].

Engineering the donor cells in order to make them produce already loaded EVs is possible also in the case of chemotherapeutic drugs and nanoparticles, as resumed in Table 4. For example, mesenchymal stromal cells are cultured for 24 h with paclitaxel and, after a change of media, cells are left to produce EVs with Paclitaxel for 48 h. These EVs can be used in the treatment of human pancreatic adenocarcinoma and they demonstrate a strong antiproliferative activity [101]. A melanoma cell line is engineered to produced EVs loaded with both survivin T34A and gemcitabine. Loaded EVs are collected and administered to pancreatic adenocarcinoma cells. The presence of survivin-T34A, which targets and inhibits survivin, an inhibitor of apoptosis, enhances the toxic effect of the Gemcitabine with lower dosages [175]. Different cell lines are incubated with methotrexate or doxorubicin and then irradiated with ultraviolet light to induce cells apoptosis. The produced ApoBDs, as delivery vehicles of chemotherapeutic drugs, exert a strong cytotoxic effect and inhibit the drug efflux from cancer cells [176]. A hybrid approach between drugs and nanoparticles involves the co-incubation of macrophages with both iron oxide NPs and a photosensitizer called m-THPC. The produced EVs containing both the two cargoes stabilize the strong hydrophobic photosensitizer drug and are injected into a mouse model. The drug allows the photodynamic therapy on cancer cells, while nanoparticles, responsive to magnetic fields, can be tracked with magnetic resonance imaging and used for hyperthermia treatments [177]. A further experiment, carried out by the previous research groups, besides the iron oxide nanoparticles, includes also a chemotherapeutic agent (doxorubicin), tissue-plasminogen activator (t-PA) and two photosensitizers (disulfonated tetraphenylchlorin-TPCS2a and 5,10,15,20-tetra(*m*-hydroxyphenyl)chlorin-mTHPC) to better enhance the antitumor ability of the produced EVs [178]. The delivery of compounds to parent cells can be difficult, especially in presence of hydrophobic molecules. For this reason, in the case of the hydrophobic photosensitizer zinc phthalocyanine, it is encapsulated in liposomes and they are used to treat the parent cells. The hydrophobic compound is secreted from the parent cells by incorporation in the EVs and then transferred to adjacent cells. This approach allows to significantly penetrates spheroids and in vivo solid tumors, enhancing the efficacy of the therapy [179]. The same procedure can be followed also for other molecule, both hydrophobic or hydrophilic, including fluorophores such as 1,1’-dioctadecyl-3,3,3’,3’-tetramethylindodicarbocyanine perchlorate (DiD) and carboxy-fluorescein, drugs (paclitaxel and tirapazamine), lipids and bio-orthogonal chemicals [180]. A similar approach is used also to incorporate nanoparticles inside EVs. Hollow-gold nanoparticles were shielded with a PEG functionalization and then incubated with human placental mesenchymal stem cells. After the uptake, the cells produced EVs loaded with hollow-gold nanoparticles. These EVs allowed to track the cell-cell communication and also perform the optical hyperthermia for cancer therapy [181].

The engineering of parent cells can also be addressed to obtain EVs loaded with molecules, such as drugs or nucleic acids, and with a specific surface functionalization (as summarized in Table 5). HEK-293T cells engineered to express Lamp2b protein, fused with a fragment of interleukin 3 (IL-3), and then incubated with Imatinib or BCR-ABL siRNA, can produce EVs loaded with the desired cargoes and expressing the IL-3 fragment on their surface. The IL-3 receptor is overexpressed in chronic myeloid leukemia and acute myeloid leukemia blasts and almost absent in hematopoietic stem cells. Exploiting this characteristic, IL-3 expressing EVs can target these cancerous cells and overcome the drug resistance to imatinib or deliver functional BCR-ABL siRNA towards imatinib-resistant cells [84]. The cell line used above can be also transfected with pDisplay vector encoding GE11 peptide or EGF, and with let-7a miRNA. The harvested EVs are functionalized with the peptide on their surface and loaded with the miRNA. Then, EVs are injected intravenously and their surface functionalization allows the specific targeting of EGFR-expressing cancer tissues, such as breast cancer. The tumor suppressor let-7a is delivered to the tumor and reduce the expression of RAS and HMGA2 inhibiting the malignant growth of cancer cells [154]. In another study, adeno-associated virus (AAV) is used as viral vector for transfection. It is broadly used for gene therapy in human, thanks to its safety profile, but it has some limitations, such as off-target gene delivery (to liver for example) and low transfection of target cells. For this reason, by transfecting the parent cells with AAV, capsids associate with the membrane and the interior part of the newly-produced EVs (called vexosomes). Harvested vexosomes show to be more resistant to anti-AAV antibodies if compared to naked AAV and they can efficiently transduce cells, enhancing gene transfer. Furthermore, parent cells are also engineered to express a transmembrane receptor on the microvesicle surface, i.e., biotin acceptor peptide-transmembrane domain (BAP-TM) receptor, allowing the specific targeting of glioblastoma cells [185]. Gene engineering method is applied to HEK-293T cell line to functionalize the CD9 tetraspanin with the RNA-binding protein HuR and then, they are modified with miR-155 or the clustered regularly interspaced short palindromic repeats (CRISPR)/Cas9 system. The produced EVs are effectively enriched by the above mentioned RNAs and in future these nanoconstructs need to be evaluated in some diseases such as liver cancer [186].

## 4. Conclusions and Future Outlook

Nanotechnology-modified EVs are promising tools for the next generation of nanomedicine for both diagnostic and therapeutic purposes with non-cytotoxic effects and a low immunogenic profiles.

In the present review, we have reported how nanoengineered EVs may be obtained either by direct post-extraction modification or by the indirect nanotechnological modification through the engineering of the parental cells producing them. From the therapeutic point of view, targeted EVs can promote the efficacy in cargo transportation toward a target cell or tissue, while also reducing off-target delivery. EVs can be loaded with very different therapeutic cargos, including both hydrophilic and hydrophobic drugs, nucleic acids like miRNA, siRNA, and recombinant proteins, or even solid-state nanoparticles. Various strategies are also available for their surface functionalization, in view of modulating the EV innate homing capabilities or refine specific targets.

Despite the advances outlined in this review, many challenges still have to be overcome to render EVs an effective and clinically-approved nanomedicine approach. First of all, achieving large-scale production of EVs for clinical use is a major challenge. In addition, a careful study on the purification processes, potentially based on immune-selection and isolation, is surely required in order to achieve high purity of the nano-engineered EVs and remove all the eventual reaction by-products or uncoupled molecules, cargos or nanoparticles after the EVs modification. Furthermore, more systematic in vivo studies are required to gain information about the re-engineered EVs toxicology, biodistribution, pharmacodynamics and pharmacokinetics.

Finally, the complex structure, the variable composition and functional activity of secreted EVs can impair their pharmaceutical approval, preventing their systematic clinical use. A potential alternative can be envisioned in the development of biomimetics EVs, thus assembled using clinical-grade and purified synthetic lipids and the necessary proteins under controlled GMP procedures to mimic the naturally-secreted ones. Strikingly, such biomimetic nanotools will not suffer from large-scale production limitations and variable compositions and can be ideal for the incorporation of many and different molecules or nanoparticles with biomodulatory, cytotoxic, anti-proliferative and imaging capabilities. Such re-engineering of EVs would thus allow novel non-immunogenic, highly stable, hemocompatible nanoplatforms, with customizable targeting and drug delivery abilities. 

However, possible drawbacks in terms of manufacture reproducibility and high cost can come against this vision. Furthermore, the precise components of natural exosomes, that are the key for obtaining efficient cell homing, therapeutic delivery and biomarker signature, are still under study and at the infancy of knowledge. 

More in general, it is thus clear that the way to efficiently obtain highly purified, well-characterized and reproducible nano-engineered EVs is still a long way. The fulfillment of these objectives will allow high performances in terms of targeting, therapeutic and diagnostic abilities, avoiding any potential side effects. Furthermore, achieving the above-mentioned vision will be the starting point of the subsequent industrial development of these novel nano-engineered EVs, including scaling up and quality control of production, rigorous pharmacokinetic and toxicological studies and, eventually, clinical testing.

## Figures and Tables

**Table 1:** EVs’ post isolation engineering: direct chemico-physical functionalizations.

EVs Type	Nanotechnological Modification	Application	Reference
Exosomes from 4T1, MCF-7 and PC3 cells	Labeling with DiR ^a^	In vivo fluorescence imaging of tumor-derived exosomes	[47]
Exosomes from 4T1 cells	Surface conjugation with azide-fluor545 by click chemistry	In vitro fluorescence imaging	[50]
Exosomes from PC12 cells	Labeling of exosomes proteins with TAMRA-NHS ^b^	In vitro fluorescence imaging	[49]
Exosomes from fetal bovine serum	PEGylation by post-insertion of DSPE-PEG-mannose or chemical conjugation of NHS-PEG	Stealth and targeted Exosomes for elevated uptake in DCs	[54]
Exosomes from embryonic stem cells	DSPE-PEG-c(RGDyK)	Targeting glioblastoma, lung cancer and prostate cancer cells	[73]
Exosomes from RAW 264.7 cells	Post-insertion of DSPE-PEG-AA	Stealth and targeted exosomes for the in vitro and in vivo treatment of lung cancer	[55]
EVs from Neuro2A cells	Post-insertion of DMPE-PEG-EGa1 nanobody	Stealth and targeted EVs for the in vitro and in vivo treatment of cancer cells	[52]
EVs from HEK 293T cells	Post-insertion of cholesterolTEG-pRNA3WJ-targeting ligands	Targeted EVs for the in vivo treatment of breast, prostate and colorectal cancer	[57]
Exosomes from MSCs	Chemical functionalization with cationized pullulan	Targeted exosomes for the in vitro and in vivo treatment of liver injury	[74]
EVs from MLP29 cells	Modification of EVs surface glycosylation by neuraminidase	Modification of EVs glycosylation for altered in vivo biodistribution	[67]
EVs from U87 and GBM8 cells	Enzymatic modification of EVs surface glycosylation and insertion of targeting ligand to DC-SIGN	Modification of EVs glycosylation and insertion of targeting ligand for improved uptake in DCs	[68]
Exosomes from Hela cells	Hexadecaarginine (R16) peptide, an arginine-rich cell-penetrating peptide	Activation of the macropinocytosis pathway, affecting cellular uptake of EVs	[75]
Exosomes from HeLa cells	Modification with LTX and GALA peptide via electrostatic interactions	Charge modified exosomes for enhanced cellular uptake and in vitro cytosolic release	[59]
Exosomes from transfected HEK 293T	Glycosylation of targeting-peptide-Lamp2b fusion proteins	Stabilization of targeting peptide-Lamp2b fusion protein with glycosylation motif	[70]
Exosomes from human colorectal carcinoma	Fe_3_O_4_ Superparamagnetic nanoparticles with high density A33 antibody	Antiproliferative effect in colorectal cancer	[71]
Extracellular vesicles from fibroblasts	Apoptotic peptide Lys-Leu-Ala (KLA) or LDL	Extravasation across BBB and target glioblastoma multiforme	[72]
Exosomes from bovine milk	Folic acid	Human lung and breast cancer reduction	[41]
Plasma membrane vesicles	Bond of the EGF ligand to the transmembrane domain of transferrin receptor	Target EGFR-expressing cancers	[76]
Exosomes-like nanoparticles from grapefruit	Inflammatory related receptor enriched membranes of activated leukocytes	Target inflammatory tumor tissues	[77]

Acronym legend: ^a^ DiR: DiIC18(7) (1,1’-dioctadecyl-3,3,3’,3’-tetramethylindotricarbocyanine Iodide); ^b^ TAMRA-NHS: carboxytetramethylrhodamine succinimidyl ester.

**Table 2:** Direct loading nanotechnological modification of EVs.

EVs Type	Nanotechnological Modification	Application	Loading Method	Reference
Exosomes from mesenchymal stem cells	Glucose-coated gold nanoparticles (NPs)	In vivo neuroimaging	Co-incubation	[78]
Exosome from lung cancer or fibroblasts	Gold NPs and doxorubicin	Lung cancer treatment	Co-incubation	[118]
EVs from breast adenocarcinoma	MOF NPs. NPs matrix contained gelonin	Inhibit adenocarcinoma growth	Sonication and extrusion	[119]
Exosomes from Hela cells	MOF NPs	Hela cells	Co-incubation	[82]
EVs from KB cells	ZnO NPs	Cytotoxic effect against KB cells	Co-incubation	[122]
EVs from endothelial, cancer and stem cell lines	Porphyrins	To improve photodynamic therapy	Electroporation, extrusion, saponin-assisted and dialysis	[97]
Exosomes from embryonic stem cells	Paclitaxel	Glioma therapy	Co-incubation	[73]
Milk-derived exosomes		To reduce paclitaxel’s side effects	Co-incubation	[102]
Exosomes from macrophages		To overcome MDR in cancer cells	Co-incubation, electroporation and sonication	[56]
Exosomes from brain cell lines		To treat brain tumor	Co-incubation	[81]
EVs from prostatic cancer		Cytotoxic effect against prostate cancer	Co-incubation	[83]
Exosomes from human colorectal carcinoma	Doxorubicin	Antiproliferative effect in colorectal cancer	Dialysis	[71]
Exosomes from breast cancer		To treat breast and ovarian cancer	Electroporation	[106]
Exosomes from breast cancer		To reduce cardiotoxicity of doxorubicin	Electroporation	[107]
Exosomes from 4T1, MCF-7, and PC3 cell line		Breast cancer	Co-incubation	[47]
Exosomes from mouse immature dendritic cells		For targeted delivery of chemotherapeutic	Electroporation	[79]
Milk-derived exosomes	Curcumin	Cervical cancer	Co-incubation	[109]
Exosomes from lymphoma cells		Activate myeloid cells in vivo	Co-incubation	[123]
Plant exosomes		Colon cancer		NCT01294072
Milk-derived exosomes	Paclitaxel, Docetaxel, Withaferin A and curcumin	Targeting and therapy of lung cancer cells	Co-incubation	[41]
Milk-derived exosomes	Celastrol	Inhibition of Hsp90 and NF-κB ^a^ activation pathways in lung cancer	Co-incubation	[124]
EVs from lung cancer	Oncolytic adenovirus and paclitaxel	Enhance immunogenicity in lung cancer	Co-incubation	[125]
Exosomes from HEK 293 cells	siRNA	Efficient delivery of siRNA in cancer cells	Electroporation	[126]
Exosomes from HEK 293 cells	Polo-like kinase 1 (PLK-1) siRNA	Silencing PLK-1 gene in bladder cancer cells	Electroporation	[127]
Exosomes from HEK 293 and MCF-7 cells	siRNA, miRNA and ssDNA ^b^	Oncogene knockdown	Sonication	[95]
Plasma-derived EVs	miRNA cel-39	Promote apoptosis of hepatocellular carcinoma	Electroporation	[128]

Acronym legend: ^a^ NF-κB: nuclear factor kappa-light-chain-enhancer of activated B cells; ^b^ ssDNA: single stranded DNA.

**Table 3:** EVs’ Surface functionalization by parent cells’ engineering.

EVs Type	Nanotechnological Modification	Application	Reference
Exosomes from breast cancer	Ac4ManNAz labeled with ADIBO-fluorescent dyes	Breast cancer imaging	[133]
Exosomes from melanoma	Gaussian Luciferase	Biodistribution and tumor targeting	[137]
Exosomes from HEK 293T			[134,135]
Exosomes from melanoma			[136]
Exosomes from HEK 293T	Alexa Fluor 680-Streptavidin	Biodistribution and tumor targeting	[135]
Exosomes from different cell lines	GFP	Monitoring and tracking of exosomes uptake in different types of cancer	[130,131,132,149]
EVs from HEK 293T	Palmitoylation signal genetically fused in-frame to the N terminus of enhanced green fluorescence protein (EGFP) and tandem dimer Tomato (tdTomato)	Monitoring the uptake by cancer cells	[150]
Exosomes from macrophage	Arginyl–glycyl–aspartic acid (RGD)-functionalized DSPE-PEG (DSPE-PEG-RGD), sulfhydryl-functionalized DSPE-PEG (DSPE-PEG-SH) and folic acid	Targeting Hela cells	[148]
EVs from squamous cell carcinoma	DSPE-PEG-Biotin and folate	Targeting breast cancer for diagnosis and therapy	[145]
Exosomes from HUVEC	DSPE-PEG-biotin	Targeting hepatocellular carcinoma	[147]
EVs from macrophage	DSPE-PEG-Biotin and folate	Targeting Hela cells	[86]
EVs from HUVEC	DSPE-PEG-Biotin	Targeting melanoma	[146]
Exosomes from HEK 293T	DARPins	Targeting HER-2 over-expressing cancer cells (breast, ovarian and gastric cancers)	[139]
Exosomes from HEK 293	Anti-HER2 scFv antibody (ML39)	Inhibit the growth of HER2 positive breast cancer	[140]
Exosomes from murine melanoma	Streptavidin-lactadherin fusion protein linked with biotinylated pH-sensitive fusogenic GALA peptide	Cancer immunotherapy	[138]
Extracellular vesicles from murine neural stem cells	Anti-EGFR fused to GPI anchor signal peptides	Targeting of Hela cells	[143]
Exosomes from dendritic cells	Lamp2b fused with iRGD (CRGDKGPDC) targeting peptide for αv integrin	Targeting breast cancer	[79]
Exosomes from dendritic cells	Carcinoembryonic antigen or HER2 linked to the C1C2 domain of lactadherin	Targeting breast cancer	[141]
Exosomes from HEK 293F cells	Prostate-specific antigen, and prostatic acid phosphatase linked to the C1C2 domain of lactadherin	Targeting prostate cancer	[142]
Exosomes from fibrosarcoma cells	Chicken egg ovalbumin by fusing it to the C1C2 domain of the lactadherin	In vivo fibrosarcoma. More efficient antitumor immune response	[151,152]
Exosomes from dendritic cell line	C1C2 domain of lactadherin is fused to soluble proteins or extracellular domain of membrane proteins	Generate antibodies against tumor biomarkers	[153]
	Transmembrane protein HLA-A2 ^a^		

Acronym legend: ^a^ HLA-A2: Human leukocyte antigen A2.

**Table 4:** Nanotechnological modification of EVs’ loading through parent cell engineering.

EVs Type	Nanotechnological Modification	Application	Reference
Human placental mesenchymal stem cells	Hollow gold NPs	Hyperthermia therapy against different type of cancer	[181]
Exosomes from hepatocellular carcinoma	Porous silicon NPs loaded with doxorubicin	Decreased the expression of multidrug-resistant protein P-glycoprotein	[182]
EVs from mesenchymal stem cells	SPIONs	Therapy against leukemia	[183]
EVs from HUVEC	Iron oxide NPs and clinical photosensitizer (Foscan)	Phototoxicity against prostate adenocarcinoma cells	[120]
Extracellular vesicles from human macrophages	Iron oxide nanoparticles and m-THPC photosensitizer	Theranostic approach against cervical and prostate cancer	[177]
Microvesicles from different cancer cell lines	A hydrophobic photosensitizer zinc phthalocyanine encapsulated in liposomes	Photodynamic therapy for different cancer cell lines	[179]
Microvesicles from human macrophages	Doxorubicin, tissue-plasminogen activator and two photosensitizers	Targeting and therapy of ovarian and prostate cancers	[178]
Exosomes from mesenchyme stromal cells	Paclitaxel	Treatment of pancreatic cancer	[101]
Exosomes from melanoma cell line	Survivin-T34A and Gemcitabine	Treatment of pancreatic adenocarcinoma	[175]
Apoptotic bodies from tumoral cells	Doxorubicin or Metotrexate	Tumor cells killing with reduce side effects	[176] NCT01854866
Exosomes from breast cancer	Curcumin	Reverse inhibition of NK cell tumor cytotoxicity in breast cancer	[110]
Exosomes from HEK 293	P53 gene	Transfer p53 gene to p53-deficient cells	[184]
Exosomes from HEK 293T	miR-21 sponges	Therapy for leukemia cells	[174]
Extracellular vesicles from breast cancer	Anti-miR-21	Theranostic method for breast cancer	[121]
Exosomes from HEK 293T	Inhibitor of miR-214	Reverse chemoresistance to cisplatin in gastric cancer	[165]
Exosomes from prostate cancer cells	Anti-miR-21 spherical nucleic acid	Prostate cancer	[155]
Exosomes from mammary carcinomas	miR-134	Increase sensitivity of breast cancers to chemotherapeutic drugs	[166]
Exosomes from mesenchyme stem cells	miR-122	Increase sensitivity of hepatocellular carcinoma to chemotherapeutic drugs	[167]
Exosomes from mesenchymal stem cells	miR-143	Inhibit migration of osteosarcoma cells	[169]
Exosomes from mesenchymal stem cells	anti-miR-9	Increase sensitivity of glioblastoma multiforme to chemotherapeutic drugs	[170]
Exosomes from mesenchyme stem cells	miR-146b	Inhibit glioma growth	[168]
Microvesicles from HEK 293T	Suicide gene mRNA and protein-cytosine deaminase fused to uracil phosphoribosyltransferase	Inhibit schwannoma tumor growth	[171]
Exosomes from HEK 293T	HGF siRNA	Inhibition of tumor growth and angiogenesis in gastric cancer	[172]
Exosomes from breast cancer cells	RAD51 and RAD52 siRNA	Gene therapy against breast cancer	[173]
Extracellular vesicles from mesenchymal stem cells	TNF-related apoptosis-inducing ligand (TRAIL)	Lung, breast, kidney cancer, pleural mesothelioma and neuroblastoma	[158]
Exosomes from chronic myelogenous leukemia cells	TNF-related apoptosis-inducing ligand (TRAIL)	Enhance apoptosis in lymphoma	[159]
Exosomes from HEK 293T	VSVG	Glioblastoma and liver cancer cells	[162]
Exosomes from lymphoblast	Nef-E7 fusion protein	T lymphocytes immune response	[161]
Exosomes from two mouse cell lines	Human MUC1 tumor antigen	Generate immune response against tumor	[157]
Microvesicles from different cancer cell lines	DiD, carboxyfluorescein, paclitaxel, tirapazamine encapsulated in fusogenic liposomes	The same cancer cell lines used to produce microvesicles	[180]

**Table 5:** EVs’ indirect modifications through combined loading and surface parent cells engineering.

EVs Type	Nanotechnological Modification	Application	Reference
Exosomes from HEK 293T	Functionalization: CD9-HuR Load: miR-155 or CRISPR/Cas9	Targeting and therapy of liver cancer	[186]
Exosomes from HEK 293T	Functionalization: Lamp2b, fused to a fragment of IL-3 Load: Imatinib or BCR-ABL siRNA	Inhibition of chronic myeloid leukemia growth	[84]
Exosomes from HEK 293T	Functionalization: GE 11 peptide Load: let-7a miRNA	Targeting and therapy of EGFR-expressing cancer tissues	[154]
Exosomes from HEK 293T cells	Functionalization: BAP-TM receptor and biotin ligase BirA Load: viral capside	Gene therapy against glioma	[185]
